# Comparison of Two New Mouse Models of Polygenic Type 2 Diabetes at the Jackson Laboratory, NONcNZO10Lt/J and TALLYHO/JngJ

**DOI:** 10.1155/2013/165327

**Published:** 2013-04-08

**Authors:** Edward H. Leiter, Marjorie Strobel, Adam O'Neill, David Schultz, Andrew Schile, Peter C. Reifsnyder

**Affiliations:** The Jackson Laboratory, 600 Main Street, Bar Harbor, ME 04609, USA

## Abstract

This review compares two novel polygenic mouse models of type 2 diabetes (T2D), TALLYHO/JngJ and NONcNZO10/LtJ, and contrasts both with the well-known C57BLKS/J-*Lepr^db^* (*db/db*) monogenic diabesity model. We posit that the new polygenic models are more representative of the “garden variety” obesity underlying human T2D in terms of their polygenetic rather than monogenic etiology. Moreover, the clinical phenotypes in these new models are less extreme, for example, more moderated development of obesity coupled with less extreme endocrine disturbances. The more progressive development of obesity produces a maturity-onset development of hyperglycemia in contrast to the juvenile-onset diabetes observed in the morbidly obese *db/db* model. Unlike the leptin receptor-deficient *db/db* models with central leptin resistance, the new models develop a progressive peripheral leptin resistance and are able to maintain reproductive function. Although the T2D pathophysiology in both TALLYHO/JngJ and NONcNZO10/LtJ is remarkably similar, their genetic etiologies are clearly different, underscoring the genetic heterogeneity underlying T2D in humans.

## 1. Introduction

The purpose of this review is to introduce two new polygenic mouse models of type 2 diabetes (T2D) and to contrast them with the most commonly studied monogenic model, the leptin receptor-deficient C57BLKS/J-*Lepr* ^*db*^/*Lepr* ^*db*^(hereafter abbreviated as *db*/*db*) mouse. On the basis of their polygenic etiologies, the absence of juvenile-onset morbid obesity, their more protracted maturity-onset development of hyperglycemia, and the absence of severe endocrine/neuroendocrine disturbances, the two polygenic models more closely reflect the phenotypes associated with the “garden variety” obesity and obesity-induced diabetes (diabesity) in humans. The genetic origins of each model will be described separately, and then their diabesity phenotypes will be compared.

## 2. The C57BLKS/J-*db*/*db* Mouse (JAX Stock 642)

 Great interest accompanied the publication of the “diabetes” mouse in 1966 [1]. A recessive mutation occurring spontaneously in the C57BLKS/J (BKS) strain produces early onset diabesity with initial moderate hyperinsulinemia followed by insulinopenia as the pancreatic islets undergo atropy due to beta-cell degeneration [1]. The mutation was identified in 1996 [2, 3] as the receptor for leptin, the adipokine hormone missing in the *ob*/*ob* (now designated *Lep* ^*ob*^) mouse [4]. Leptin sensing in hypothalamic nuclei is essential for normal regulation of satiety as well as multiple metabolic and neuroendocrine/reproduction pathways. Hence, the extreme leptin resistance produced by the absence of an intracellular signaling domain in leptin receptor of the *db*/*db* mouse produces hyperphagia and morbid obesity, reproductive failure, and severe insulin resistance. The juvenile-onset of hyperglycemia (within a week of weaning onto carbohydrate-containing chow), coupled with the massive beta-cell loss and early mortality as hyperglycemia progressed, suggested an amalgam between type 2 and type 1 diabetes. Although the origin of the diabesity is clearly monogenic, the development of severe diabetes is dependent upon multiple other background genes. The same mutation transferred onto the related C57BL/6J (B6) background produces an even more severe insulin resistance obesity syndrome, but not chronic, severe diabetes. The BKS strain is a black-pigmented strain representing an admixture of B6 and, apparently, DBA/2 genomes [5]. In the latter strain, *db* mutation homozygosity produces severe diabetes with high mortality, like BKS [6]. The identification of these genetic background modifiers has yet to be established. The *db*/*db* model has been widely used in the evaluation of antiobesity and antidiabetic compounds and therapies because the penetrance of the mutation is almost 100% (e.g., all mutants become reproducibly obese and diabetic at an early age). Further, mutants of both sexes develop diabesity and either the lean littermates from heterozygous matings (designated +/+ or +/*db*) or standard BKS mice can be used as controls. The mutant mice are not particularly stress sensitive such that pharmaceuticals can be administered by gavage when necessary. Hence, this mouse model has remained a mainstay for pharmaceutical efficacy testing. An extensive bibliographic listing of phenotypic and husbandry information, as well as a photograph of the obese mutant mouse and a lean control, may be found at http://jaxmice.jax.org/strain/000642.html.

## 3. The NONcNZO10/LtJ Male Mouse (JAX Stock 4456)

The nomenclature descriptor denotes a recombinant congenic strain comprising approximately 88% genome contribution from the NON/LtJ strain (JAX Stock 2423) and 12% from the New Zealand obese (NZO/HlJ, JAX Stock 2105) strain. NZO/HlJ mice of both sexes develop polygenic, morbid obesity with marked insulin resistance. However, the leptin-leptin receptor axis appears intact, such that leptin resistance is peripheral, not central [7]. As in BKS-*db*/*db* mice, NZO/HlJ males show postpubertal development of hyperleptinemia, hyperinsulinemia, thermoregulatory defects, hypertension, and impaired glucose tolerance [8, 9]. Unlike the monogenic obesity of BKS-*db/db* mice, NZO obesity is under complex polygenic control. Moreover, diabesity development is male sex limited, maturity onset, and represents a threshold phenomenon predicated on early rate of weight gain [9]. Although NZO/HlJ mice outbreed well, breeding within strain is poor. 

The polygenic NONcNZO10/LtJ model (hereafter abbreviated as NcZ10) was specifically developed at The Jackson Laboratory over the past decade to circumvent the extreme NZO phenotypes shared with BKS-*db/db* mice and that distinguished both from human “garden variety” obesity while at the same time increasing the frequency of male hyperglycemia development and improving within-strain reproduction. The NON/LtJ strain was used as a “genetic scaffold” on which to build the new model. This strain was selected in Japan for high nonfasting blood glucose [10] and unpublished studies at The Jackson Laboratory showed that NON/LtJ males were markedly more sensitive to high fat diet induced obesity than B6 males. The strategy used was to outcross agouti NZO and albino NON mice and identify by genetic segregation analysis in the F2 and first backcross generations the quantitative trait loci (QTL) contributing both to obesity and diabesity. At least 10 codominant or additive polygenes capable of complex epistatic interactions and derived from both parental genomes were identified [11]. This genetic information was used to transfer as many diabesity QTL as possible onto the NON genetic background at a second backcross, followed by inbreeding to fix these QTL to homozygosity [11]. The result, as will be seen below, was creation of an albino strain that reproduced well, exhibited a more moderate obesity than NZO, and exhibited a male diabesity frequency of 90–100%. A genetic control strain, NONcNZO5/LtJ (JAX Stock 4455), was developed to contain the reciprocal (diabesity resistance) QTL of those in NcZ10 [12]; unfortunately, this obese but diabetes-free strain exhibits the poor reproduction characteristic of NZO/HlJ. Thus, the NON/LtJ or other related Swiss-derived albino inbred strains (SWR/J, SJL/J, FVB/NJ, and ICR/HaJ) are suggested as nonobese controls for physiologic comparisons. When NON/LtJ is used, it must be kept on a low fat diet to avoid diet induced obesity. As will be demonstrated below, NcZ10/LtJ males must be placed on an elevated fat diet for diabesity to develop.

 Hyperinsulinemic-euglycemic clamping demonstrated insulin resistance in liver and skeletal muscle in prediabetic 8-week-old males prior to later increases in hepatic lipid content. The muscle resistance was associated with reduced GLUT4 protein, but not IRS-1 [13]. Livers of chronically hyperglycemic males show moderate to severe hepatosteatosis on a chow diet containing 6% fat [12]. Islet histopathology in NcZ10 males shows an early islet hypertrophy followed by beta-cell degranulation and beta-cell atrophy as hyperglycemia becomes chronic [11, 12]. After transfer of the colony from a research colony at The Jackson Laboratory to a high barrier production facility on campus, a 6% fat containing chow diet was found to no longer support attainment of the early body weight gain thresholds required for diabesity development. Raising the fat content of the chow to 10-11% restored diabesity development to expected frequencies. The reason for the higher dietary fat requirement for diabesity is assumed to be a more refined enteric flora in the high barrier colony. Additional phenotypic information, husbandry details, bibliographic information, and a photograph of the mouse may be found on the web at http://jaxmice.jax.org/strain/004456.html.

## 4. TALLYHO/JngJ Male Mice (JAX Stock 5314)

TALLYHO/Jng (hereafter abbreviated TH) males represent another recently developed polygenic T2D model at The Jackson Laboratory that also expresses less extreme diabesity phenotypes more consistent with the common forms of human T2D. This albino strain derives from the progeny of two diabetic males discovered in an outbred colony of Theiler's original mice in the United Kingdom and imported to The Jackson Laboratory in 1992 [14]. Inbreeding with selection of progeny of diabetic males has produced a polygenic diabesity model that is very similar to NcZ10 in many respects. However, the combination of polygenes interacting to produce diabesity in TH, for the most part, appears distinct from in NcZ10, attesting to the genetic heterogeneity that clearly must also underlie a very common disease in a very genetically heterogeneous human T2D population. Outcross of TH to B6 identified multiple obesity and diabesity QTL, with a major hyperglycemia QTL on chromosome 19 and a hyperlipemia QTL on chromosome 1 (reviewed in [14]). Pancreatic islet histopathology of diabetic TH males also shows early islet hypertrophy/hyperplasia followed by beta-cell degranulation and some beta-cell loss [15]. Hyperinsulinemic-euglycemic clamping indicated peripheral insulin resistance at 10 weeks of age in prehyperglycemic males, with hepatic insulin resistance developing with hyperglycemia at 16 weeks of age [16]. Insulin resistance in white adipose tissue was associated with reduced GLUT-4 cycling and increased IRS-1 degradation [17]. Significant heart diastolic dysfunction (Sartoretto et al., 2010) [18] and cerebral vascular anomalies [19] were documented in diabetic TH males. This strain is characterized by age-associated bilateral hydronephrosis in both sexes (Dr. J. K. Naggert and Y. Wang, personal communication). Additional phenotypic information, husbandry details, bibliographic information, and a photograph of the mouse may be found on the web at http://jaxmice.jax.org/strain/005314.html.

## 5. Comparison of New Polygenic Models to the Monogenic BKS-*db/db* Model

**Figure 1:**
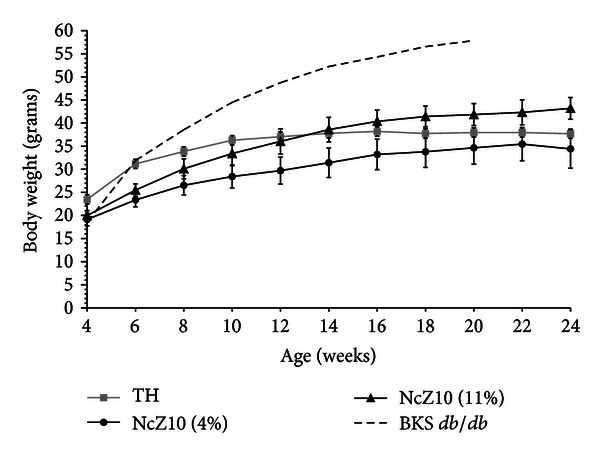
Comparative weight gains of groups of 20 BKS-*db/db* and TH males fed standard 6% fat-containing chow, and the same number of NcZ10 males fed either 4% or 11% fat-containing chow. Data are means ± SD.

**Figure 2:**
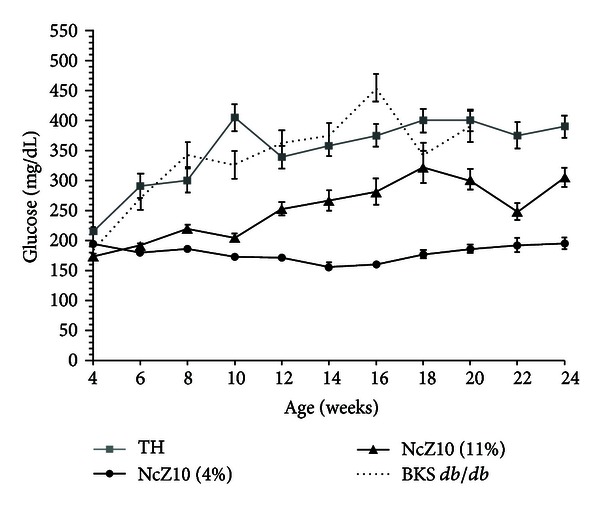
Development of hyperglycemia (defined as nonfasting serum glucose ≥250 mg/dL) in the same cohorts shown in Figure 1. Data are means ± SEM.

**Figure 3:**
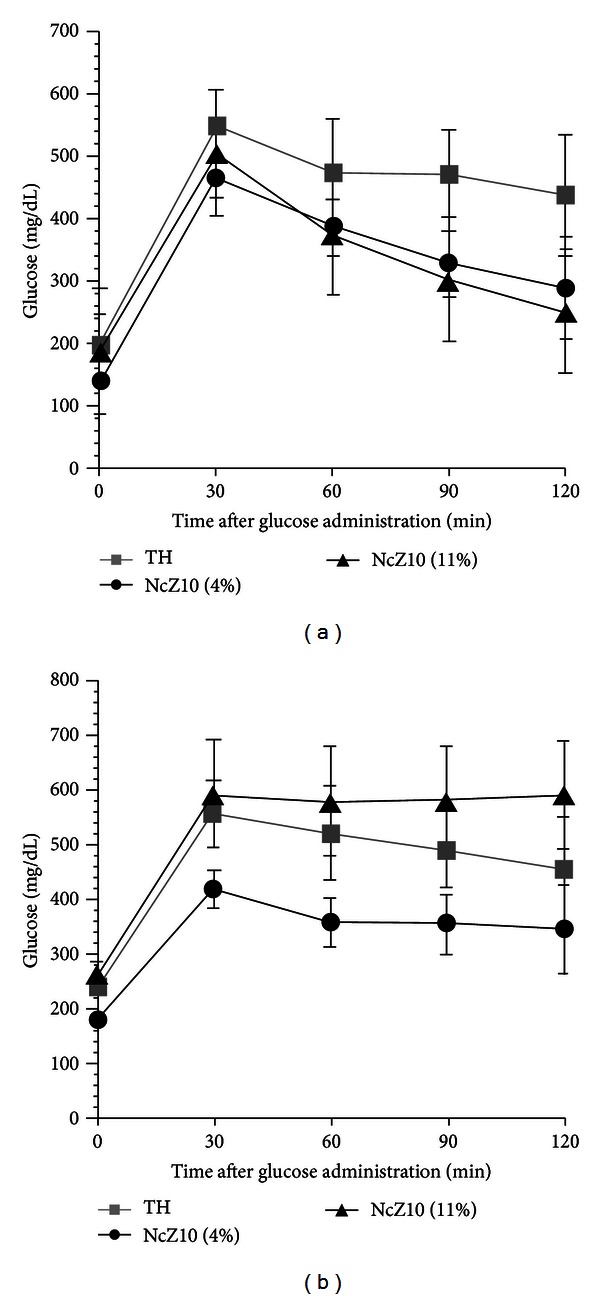
Oral glucose tolerance tests (2 g glucose/kg) of TH and NcZ10 males (a) at 8 weeks of age and (b) at 24 weeks of age (*n* = 10/group).

Body weight gain data in Figure 1 contrasts the rapid juvenile-onset weight gain and eventual morbid obesity in BKS-*db/db* males versus the more moderate and maturity-onset weight gains in TH and NcZ10 males. Data in Figures 1 and 2 also demonstrate the unusual sensitivity of the NcZ10 strain to the diabetogenic action of dietary fat. As seen in Figure 2, the significantly slower and lower weight gains experienced by NcZ10 males fed the 4% fat-containing chow diet failed to allow them to achieve the degree of obesity necessary to trigger development of hyperglycemia (defined here as a nonfasting blood glucose of >250 mg/dL). Although the TH males fed a 6% fat-containing chow diet showed an initial high weight gain within a week of weaning, this rate moderated after puberty and was comparable to NcZ10 males, but much lower than BKS-*db/db* males. The cohort of TH males aged at The Jackson Laboratory on a 6% fat-containing chow diet showed an earlier onset of hyperglycemia than reported in the literature (10–12 weeks), with glucose values over time similar to the progressive rise seen in BKS-*db/db* males. In the NcZ10 male cohort fed the 11% fat-containing chow, chronic hyperglycemia was only established at 12 weeks. Notably, hyperglycemia did not develop in the 4% fat-containing chow-fed males (Figure 2) that failed to attain the requisite body weight thresholds for diabesity development. Oral glucose tolerance data in Figure 3 show that TH and NcZ10 males develop glucose intolerance at different rates. TH males already exhibited impaired glucose tolerance at 8 weeks compared to age-matched NcZ10 males fed either 4% or 11% fat-containing chow (Figure 3(a)). At 24 weeks of age, chronically diabetic TH and NcZ10 males fed the 11% fat chow exhibited comparable, severe impairment in oral glucose tolerance (Figure 3(b)). In nondiabetic 24-week-old NcZ10 males maintained on the 4% fat-containing chow (Figure 3(b)), glucose tolerance had deteriorated, but impairment was still significantly less severe than for either group of diabetic males.

Comparative clinical chemistries of the two polygenic models also distinguish them from the BKS-*db/db* model (Table 1). Circulating concentrations of insulin and leptin for all three models are available from the literature. Plasma insulin content in the BKS-*db/db* model shows temporal changes. Marked hyperinsulinemia (mean ~55 ng/mL at 6 weeks) develops in the early phase of the syndrome as the islet mass undergoes initial hyperplasia and beta cells hypersecrete insulin [20]. However, after 8 weeks of age, plasma insulin drops sharply to normal or subnormal concentrations (≤1 ng/mL) as beta-cell loss and islet atrophy occur [20]. In contrast, the combination of permanent adiposity coupled with deficient leptin receptor signaling in BKS-*db/db* produces a permanent, severe hyperleptinemia, with concentrations reaching >200 ng/mL [21]. Published plasma insulin and leptin concentrations for both NcZ10 and TH contrast sharply with BKS-*db/db* while being very comparable to each other. Published plasma insulin for hyperglycemic 24-week-old NcZ10 males was 6 ng/mL, only slightly higher than the 4 ng/mL in normoglycemic NON/Lt control males. Similarly, leptin concentration (17.5 ng/mL) was only 2-fold higher than the NON/Lt parental control and at least 3-fold lower than the 60 ng/mL reported in morbidly obese NZO parental males at the same age [12]. Published values for plasma insulin in diabetic 16-week-old TH males closely reflect those reported for NcZ10 males, with insulin reaching peak values of 12 ng/mL at 12 weeks and remaining elevated at 16 weeks [15]. Plasma leptin values in TH males are reported to be variable, but modestly elevated compared to normal B6 males [14]. Thus, both polygenic models differ from both the monogenic obesity in BKS-*db/db* males and the polygenic obesity in NZO males with only moderate elevations in these two endocrines. 

Dyslipidemia characterized by marked hypertriglyceridemia on a standard chow diet has been reported in diabetic males in all three models. However, despite the finding of significantly elevated total cholesterol and nonesterified free fatty acids (NEFA) in the 20-week-old BKS-*db*/*db* male cohort from The Jackson Laboratory's production colony, we were unable to replicate a significant elevation in serum triglycerides compared to lean BKS-*m*+/*m*+ male controls (Table 1). Given a previous study reporting a plasma triglyceride value of 400 ± 19 mg/dL for diabetic BKS-*db*/*db* males at this age [22], we cannot explain the difference. In contrast, data in Table 1 do confirm the literature reporting marked hypertriglyceridemia in both polygenic models, with TH mean concentration significantly higher than in NcZ10 males. High hemoglobin A1c concentrations are present at 20 weeks of age in blood of BKS-*db/db*, TH, and NcZ10 males (the latter fed the 11% fat chow). NcZ10 males fed the 4% fat-containing diet, although not overtly hyperglycemic at 24 weeks, exhibited plasma triglycerides as high as hyperglycemic males fed the 11% fat chow (data not shown). Thus, in the NcZ10 model, the dyslipidemia develops independently of chronic hyperglycemia. Hepatic steatosis has been described in both diabetic BKS-*db/db* [21] and NcZ10 males [12] and is also present in diabetic TH males (Dr. J. K. Naggert, personal communication). The significantly higher alanine aminotransferase concentrations in serum of BKS-*db*/*db* males may reflect not only increased liver pathology, but also ongoing endocrine pancreatic tissue destruction.

## 6. Discussion

The phenotypes of the diabesity syndromes developing in the two new polygenic models, NcZ10 and TH, are very similar to each other despite the distinct polygenetic etiologies. Although the dyslipidemias in each are very comparable to those of the BKS-*db/db* mouse, the new polygenic models are clearly differentiated from the latter by the absence of uncontrolled hyperphagia. Further, less severe disturbances in the hypothalamic-pituitary-adrenal axis in the polygenic models are evidenced by their retention of normal reproductive function. Finally, the maturity-onset nature of hyperglycemia coupled with the less extreme plasma concentrations of insulin and leptin makes the polygenic models much more reflective of the most common forms of human T2D.

An obvious advantage of a monogenic diabesity model is the availability of a wild-type genetic control not available in a polygenic model. In both TH and NcZ10 strains, females do not develop hyperglycemia and thus can be used for comparison to the effects of hyperglycemia in males. As shown in this review for the NcZ10 model, manipulation of either the total amount of dietary fat or its composition [23] can be used to provide normoglycemic controls. Likewise, normoglycemic NONcNZO5LtJ males are matched with NcZ10 for obesity, but have a less diabetogenic adiposity [24]. Initially, these two stocks were phenotyped on a 6% fat-containing chow diet that was sufficient for diabesity development in NcZ10 in a low barrier research colony at The Jackson Laboratory. However, this diet failed to support requisite weight gains for diabesity development after rederivation of NcZ10 into a high barrier production colony. The need to increase total dietary fat to reconstitute diabesity is assumed to reflect a more refined enteric flora of mice in the production colony phenotyped for this review. The use of dietary manipulation to generate normoglycemic controls for TH males has not yet been reported; possibly high fat and carbohydrate-free diet demonstrated to block diabesity in the NZO diabesity model [25] may generate such controls.

Given the phenotypic similarities of the diabesity syndromes in TH and NcZ10 males, it is perhaps not surprising that both more closely replicate the defects in wound healing that characterizes diabetic human patients than does the BKS-*db/db* model [26, 27]. Differences do distinguish the two polygenic models in their responses to antidiabetic drugs administered by gavage. The Jackson Laboratory tested the responses of diabetic males from all 3 models to the gavage-administered thiazolidinedione, rosiglitazone. This compound fed in the chow diet had previously been shown to exert potent antihyperglycemic action in diabetic NcZ10 males [28]. However, when treatment entailed multiple gavages, NcZ10 males unlike both the BKS-*db*/*db* and TH diabetic models in which antihyperglycemic responses were limited to drug-treated recipients showed a strong placebo response to the vehicle. Shipment-associated weight loss and consequent failure to achieve requisite thresholds for diabesity development [11] have characterized some shipments of prediabetic NcZ10 males, but not TH males from the production facility of The Jackson Laboratory. The basis for this enhanced stress sensitivity affecting penetrance of the diabesity phenotype of the NcZ10 compared to the TH model remains to be elucidated.

## 7. Summary and Conclusions

The newer TH and NcZ10 models are important additions to mouse models of T2D. The major features distinguishing these two polygenic obesity/diabesity models from the better-known BKS-*db/db* model are summarized in Table 2. 

TH and NcZ10 males more accurately model the human condition in terms of the polygenic basis for their obesity syndromes, coupled with their maturity onset development of hyperglycemia without the extreme disruptions in neuroendocrine pathways associated with mutations in either the leptin or leptin receptor genes. Because they have become available only quite recently, the TH and NcZ10 polygenic diabesity models are not that well known to the diabetes research community. Clearly, much more research on both models is warranted. Although the T2D pathophysiology in both TH and NcZ10 is remarkably similar, their genetic etiologies are clearly different [12, 29]. This certainly models the situation in human T2D, wherein genetic analysis of patients with common disease pathophysiology reveals complex interactions between a large and variable number of genes and the environment. The newer models should certainly not be considered as replacements of the monogenic models such as BKS-*db/db* which has become a reference strain for drug testing. Given the genetic heterogeneity underlying T2D in humans [30, 31], the newer models discussed in this review should not supplant the “tried and true” older models, but rather extend them. A new drug that shows efficacy without toxicity in one model may or may not prove widely therapeutic in a genetically heterogeneous human T2D patient population. However, if the same drug shows equal efficacy in multiple available models, each with a distinct genetic underpinning, confidence that the drug would safely and effectively treat a broader spectrum of human patients surely is increased. 

## Figures and Tables

**Table 1:** Comparison of selected serum analytes in the male cohorts shown in the figures. Data show mean ± SEM for 8–10 males at 20 weeks of age. Lean age-matched BKS males are shown for comparison. Groups were maintained on 6% fat chow except for NcZ10 males that were fed 11% fat chow. Hemoglobin A1c (HbA1c) values are shown to document chronic hyperglycemia in the 3 diabetic stocks.

Strain	Glucose mg/dL	HbA1c % NGSP units	Triglyceride mg/dL	Total cholesterol mg/dL	HDL cholesterol mg/dL	Nonesterified fatty acids mEq/L	ALT^f^ (IU/L)
NcZ10	475 ± 30	10.4 ± 0.2	255 ± 12^e^	128 ± 4^e^	97 ± 3	1.90 ± 0.08^e^	44.2 ± 1.1
TH	520 ± 36	9.0 ± 0.5	491 ± 57	151 ± 3	106 ± 3	2.47 ± 0.09	44.6 ± 4.1
BKS-*db/db *	444 ± 48	12.8 ± 0.8^d^	141 ± 12^b^	163 ± 6	127 ± 5^d^	2.82 ± 0.17	125.1 ± 9.8^f^
BKS-*m*+/*m*+^a^	167 ± 14^c^	5.7 ± 0.2^c^	131 ± 7^b^	98 ± 6^c^	71 ± 2^c^	1.92 ± 0.08	39.2 ± 5.3

^a^ *m*+/*m*+ from the cross with the *db* gene in repulsion with the misty (*m*) gene.

^b^ *db/db* and lean control significantly lower (*P* ≤ 0.05) than TH and NcZ10.

^c^Lean control significantly lower (*P* ≤ 0.05) than all 3 diabetic stocks.

^d^ *db/db* significantly higher (*P* ≤ 0.05) than TH and NcZ10.

^e^NcZ10 significantly lower (*P* ≤ 0.05) than TH and *db/db*.

^f^Alanine aminotransferase; *db/db* significantly higher (*P* ≤ 0.05) than control, TH, and NcZ10.

**Table 2:** Summary comparison of the 3 models.

	BKS-*db/db *	NONcNZO10/LtJ	TALLYHO/JngJ
Obesity	Monogenic, morbid	Polygenic, moderate	Polygenic, moderate
Diabetes	Juvenile onset	Maturity onset	Maturity onset
Leptin resistance	Central, total	Peripheral, moderate	Peripheral, moderate
Insulin resistance	Severe	Moderate	Moderate
Sex bias	None	Males	Males
Littermate controls	Yes	No	No
Fertile	No	Yes	Yes
